# Rapid Evolution of Glycan Recognition Receptors Reveals an Axis of Host–Microbe Arms Races beyond Canonical Protein–Protein Interfaces

**DOI:** 10.1093/gbe/evad119

**Published:** 2023-06-30

**Authors:** Zoë A Hilbert, Paige E Haffener, Hannah J Young, Mara J W Schwiesow, Ellen M Leffler, Nels C Elde

**Affiliations:** Department of Human Genetics, University of Utah, Salt Lake City, Utah, USA; Howard Hughes Medical Institute, University of Utah School of Medicine, Salt Lake City, UT, USA; Department of Human Genetics, University of Utah, Salt Lake City, Utah, USA; Department of Human Genetics, University of Utah, Salt Lake City, Utah, USA; Howard Hughes Medical Institute, University of Utah School of Medicine, Salt Lake City, UT, USA; Department of Human Genetics, University of Utah, Salt Lake City, Utah, USA; Howard Hughes Medical Institute, University of Utah School of Medicine, Salt Lake City, UT, USA; Department of Human Genetics, University of Utah, Salt Lake City, Utah, USA; Department of Human Genetics, University of Utah, Salt Lake City, Utah, USA; Howard Hughes Medical Institute, University of Utah School of Medicine, Salt Lake City, UT, USA

**Keywords:** host–pathogen interactions, evolutionary conflict, rapid evolution, balancing selection, pattern recognition receptor, microbial glycans

## Abstract

Detection of microbial pathogens is a primary function of many mammalian immune proteins. This is accomplished through the recognition of diverse microbial-produced macromolecules including proteins, nucleic acids, and carbohydrates. Pathogens subvert host defenses by rapidly changing these structures to avoid detection, placing strong selective pressures on host immune proteins that repeatedly adapt to remain effective. Signatures of rapid evolution have been identified in numerous immunity proteins involved in the detection of pathogenic protein substrates, but whether similar signals can be observed in host proteins engaged in interactions with other types of pathogen-derived molecules has received less attention. This focus on protein–protein interfaces has largely obscured the study of fungi as contributors to host–pathogen conflicts, despite their importance as a formidable class of vertebrate pathogens. Here, we provide evidence that mammalian immune receptors involved in the detection of microbial glycans have been subject to recurrent positive selection. We find that rapidly evolving sites in these genes cluster in key functional domains involved in carbohydrate recognition. Further, we identify convergent patterns of substitution and evidence for balancing selection in one particular gene, *MelLec*, which plays a critical role in controlling invasive fungal disease. Our results also highlight the power of evolutionary analyses to reveal uncharacterized interfaces of host–pathogen conflict by identifying genes, like *CLEC12A*, with strong signals of positive selection across mammalian lineages. These results suggest that the realm of interfaces shaped by host–microbe conflicts extends beyond the world of host–viral protein–protein interactions and into the world of microbial glycans and fungi.

**Significance**

The impact of host–pathogen conflicts in driving evolutionary innovation in mammalian immune proteins is well documented; however, the role of nonprotein components of microbial pathogens in contributing to such evolutionary processes is not well understood. We identify widespread signals of adaptive evolution in mammalian immune receptors that engage largely with carbohydrate components that decorate the outer surfaces of diverse microbial pathogens, from viruses to fungi. Further, we demonstrate how interactions involving nonproteinaceous components of microbes have driven evolutionary change in mammalian genes across multiple timescales, including evidence for balancing selection in a fungal melanin receptor gene in many human populations. Collectively, these findings extend the realm of host–microbe evolutionary conflicts beyond traditionally studied protein–protein interfaces and demonstrate the impressively broad impact microbes have on the evolution of their animal hosts.

## Introduction

Recognition of microbial pathogens by mammalian immune proteins is essential for activation of protective immune responses and organismal survival. Pattern recognition receptors (PRRs) encompass a diverse group of host proteins which are integral in detecting microbial pathogens as foreign invaders through recognition of unique molecular features (Medzhitov 2007; Kawai and Akira 2008; Vance et al. 2009; Tan et al. 2015). These pathogen-associated molecular patterns are similarly as diverse as the receptors that they engage with and range from proteins, like bacterial flagellins, to nucleic acids, to complex carbohydrates, or glycans.

Microbial glycans are a defining feature of the cell walls of bacteria and fungi and decorate the outer membranes and surfaces of parasites, whereas glycosylation of coat and surface proteins is also well documented in many viruses (Nyame et al. 2004; Comstock and Kasper 2006; van Kooyk and Rabinovich 2008; Raman et al. 2016; Gow et al. 2017). Glycan-recognizing PRRs include, among others, a subset of the Toll-like receptors (TLRs) as well as many members of the calcium-binding C-type lectin receptor (CLR) family. Although the specific glycans recognized by some of these PRRs are known—such as Dectin1's affinity for ß-glucans or TLR4's for lipopolysaccharide—for many of these receptors, the exact molecular patterns on microbial surfaces required for recognition are unclear, as is the extent to which variation of these patterns among different microbial species might affect recognition (Poltorak et al. 1998; Brown and Gordon 2001; Herre et al. 2004; Park et al. 2009; Werling et al. 2009).

Phylogenetic analysis of immune genes, including PRRs, has revealed them to be among the most rapidly evolving genes in mammalian genomes, reflecting the pace of evolution needed to keep up with constantly shape-shifting pathogens (George et al. 2011; Daugherty and Malik 2012; Rausell and Telenti 2014; Wang and Han 2021). Studies of rapidly evolving immune genes in mammals have largely focused on genes involved in interactions with pathogen-produced protein factors. Comparative analyses of recurrent rapid evolution (or positive selection) on the amino acid level frequently reveal the consequential interaction interfaces between host and pathogen proteins. Related experimental studies show how evolution on both sides of these interactions can have functional implications for both host and pathogen (Sawyer et al. 2005; Elde et al. 2009; Mitchell et al. 2012; Barber and Elde 2014; Tenthorey et al. 2020; Carey et al. 2021). These studies reveal the extent to which microbes can spur diversification and evolutionary innovation in the hosts they infect. However, detection of these host–pathogen “arms races” has so far been primarily limited to protein–protein interfaces involving viruses and bacteria, even though engagement between hosts and infectious microbes involves a wide variety of biological macromolecules and species.

Fungi, in particular, represent a major class of human pathogens which are currently auspiciously absent from studies of host–pathogen evolutionary conflict. Systemic fungal infections are associated with severe disease and high mortality rates in human patients and the emergence of multidrug resistant strains has increased dramatically in recent years (Fisher et al. 2022). Beyond human patients, fungal infections pose a severe threat to the health of food crops, and fungal pathogens are currently responsible for massive declines in amphibian and hibernating bat populations world-wide (Fisher et al. 2020). Despite the importance of these pathogens for the health of evolutionarily diverse organisms, our understanding of the role of host–fungal conflicts in shaping vertebrate immune defenses has been hampered by the relative lack of known protein-based fungal virulence factors.

As the first line of defense against recognition by host immune factors, diversification in microbial cell wall components and organization has been well documented in bacterial and fungal pathogens (Gow et al. 2017; Imperiali 2019). Further, molecular mimicry of host glycan structures, such as sialic acids, and hijacking of glycosylation pathways has been demonstrated to be a common mechanism of immune evasion in numerous pathogenic bacteria and viruses (Comstock and Kasper 2006; Vigerust and Shepherd 2007; Carlin et al. 2009; Varki and Gagneux 2012; Raman et al. 2016). Although glycan hijacking and mimicry in fungi is less well documented, reports of sialic acids and sialoglycoconjugates in the cell walls of several fungal species, including the pathogenic species *Candida albicans* and *Cryptococcus neoformans*, suggest that fungi may also use methods of molecular mimicry to evade host immune recognition (Rodrigues et al. 1997; Soares et al. 2000; Masuoka 2004). And in fungi, regulated secretion of exopolysaccharide “decoys” correlates with decreased immune infiltration, suggesting these microbes have developed numerous strategies to prevent their recognition by host immune systems (Denham et al. 2018).

Such evasion strategies among microbes suggest the potential for selective pressures to exist on immune receptors to be able to maintain the ability to recognize microbial glycans and initiate immune responses to control infection. In this study, we identify signatures of positive selection in a set of primarily glycan-recognizing PRRs across three distinct mammalian lineages, suggesting that host–pathogen interfaces involving nonproteinaceous macromolecules may represent a new dimension of host–microbe arms races and can spur evolution in all species involved.

## Results

### Signatures of Rapid Evolution Are Pervasive Among Mammalian CLRs and Other Carbohydrate Recognition PRRs

**Fig. 1.**
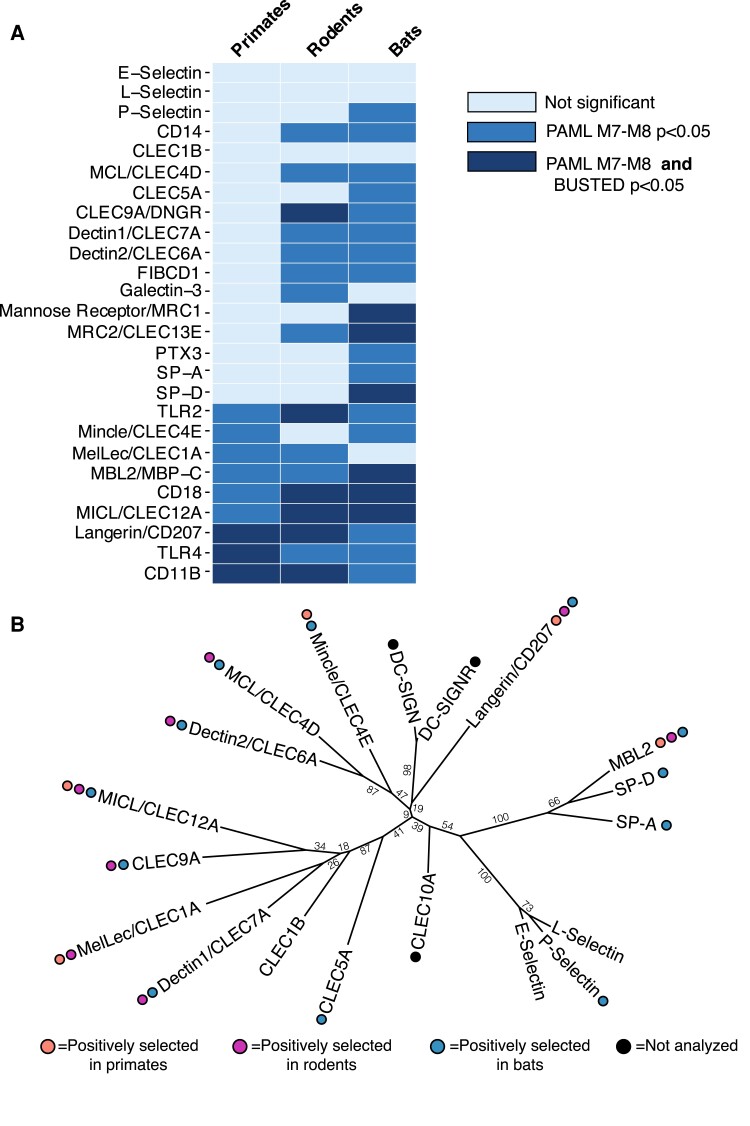
Positive selection across mammalian carbohydrate recognition PRRs. (*A*) Positive selection analyses of 26 glycan PRRs in primates (left column), rodents (middle), and bats (right column). Colored boxes indicate whether evidence of positive selection was supported by PAML analyses only (medium blue) or by both PAML and BUSTED analyses (dark blue). Genes with no evidence for positive selection are represented by pale blue boxes. Statistical cutoffs were *P* < 0.05 for PAML M7 versus M8 likelihood ratio tests and for BUSTED analysis. (*B*) Patterns of positive selection mapped onto a phylogenetic tree of the human CTLD domains. Only genes from the gene set with CTLDs are represented. Colored circles represent evidence of positive selection in the primate (orange), rodent (purple), and/or bat (blue) lineages. Genes with black circles were not analyzed in this study because of unclear ortholog relationships across mammals but do have important roles in pathogen detection in mammals. Numbers indicate bootstrap values from phylogenetic tree construction using IQ-TREE.

To assess whether host genes involved in microbial carbohydrate recognition are rapidly evolving in mammals, we compiled a list of 26 relevant genes for analysis (fig. 1*A* and *B* and Supplementary material online). These genes were selected based on annotated functions in the recognition of microbial cell walls or other carbohydrate components of microbial cells. Genes were also prioritized for analysis based on documented expression patterns. Namely, genes expressed by immune cells or on mucosal surfaces were prioritized given their relevance for interactions with microbes and defense against infection.

More than half of the selected PRR genes contain an annotated C-type lectin domain (CTLD), including a number of CLR family members with a single CTLD (e.g., *Dectin1/CLEC7A*, *Langerin/CD207/CLEC4K*, *Mincle/CLEC4E*) as well as the soluble CTLD-containing proteins (*MBL2*, *SP-A*, *SP-D*) and the multiple CTLD-containing mannose receptors (*MRC1* and *MRC2*). Beyond the CLRs and other CTLD-containing proteins, our list also included a putative chitin receptor (*FIBCD1*), complement receptor 3 (*CD11B*/*CD18*), and TLRs (*TLR2* and *TLR4*). Among this latter group, there have been previous reports of signatures of positive selection in the TLR genes as well as *CD11B*, which we were able to replicate in this study, while also extending analyses of selection in these genes to additional mammalian lineages (Wlasiuk and Nachman 2010; Areal et al. 2011; Liu et al. 2019; Boguslawski et al. 2020; Judd et al. 2021). Finally, we also included in our analyses the CTLDs of three conserved mammalian selectin genes: *E-Selectin, L-Selectin*, and *P-Selectin*. These CTLD containing proteins are expressed on a variety of different cell types and act to coordinate cell adhesion and leukocyte trafficking through recognition of “self”-produced carbohydrate ligands or self-associated molecular patterns (SAMPs) (Varki 2011; Cummings et al. 2022). Given their important role in recognition of these SAMPs on leukocytes and other mammalian cells and no documented role in the recognition of microbes, we hypothesized that the CTLDs from these Selectin genes would not be subject to the same evolutionary pressures as other candidate genes involved in direct interactions with infectious microbes.

For each of these genes, we obtained orthologous sequences from publicly available databases for species within three distinct mammalian lineages: simian primates, mouse-like rodents (*Myomorpha*), and bats. Primates were chosen given their relevance to human health, whereas bats and rodents have been implicated as important reservoirs for many microbial species with zoonotic potential, suggesting that such evolutionary analysis may reveal unique patterns of selection among PRRs across these three mammalian lineages (Han et al. 2015; Guth et al. 2022). The orthologous gene sequences within each lineage were aligned and each gene was assessed for signals of recurrent positive selection using a combination of different analysis algorithms, including Phylogenetic Analysis by Maximum Likelihood (PAML) and Branch-Site Unrestricted Test for Episodic Diversification (BUSTED) in the Hypothesis Testing using Phylogenies (HyPhy) suite (Pond et al. 2005; Yang 2007; Murrell et al. 2015). Both algorithms use the calculation of the ratio of the nonsynonymous to synonymous substitution rates (dN/dS) and model fitting comparisons in order to make inferences about signatures of selection across genes and phylogenies. For genes or codons under purifying selection, nonsynonymous substitutions are selected against, leading to dN/dS values less than 1. In contrast, positive selection—or rapid evolution—is characterized by the relative enrichment of nonsynonymous substitution rates, which can be identified by elevated dN/dS values (>1) in these genes or at specific codons within genes.

Using the site models implemented in PAML along with BUSTED, we identified signatures of site-specific positive selection by at least one of the two algorithms (BUSTED *P* < 0.05 or PAML M7 vs. M8 likelihood-ratio test [LRT] *P* < 0.05) in nine (35%) of the primate PRRs (fig. 1 and supplementary data file 1, Supplementary Material online). This number was strikingly elevated among the rodent and bat lineages, with 16 (62%) and 21 (81%) genes under positive selection in these groups, respectively. Mapping these positively selected genes onto a phylogenetic tree of the CTLDs from the CLR-type PRRs revealed no clear pattern to the distribution of positive selection across this family of receptors (fig. 1*B* and supplementary fig. S1*B*, Supplementary Material online). Instead, rapid evolution seems pervasive across the entire family of CLRs that were analyzed.

Through these approaches, we identified a core set of six PRRs predicted to be under positive selection by one or both algorithms in all mammalian lineages tested. These core genes include those, such as *TLR4*, with long-established roles in microbial recognition and previously defined ligands. However, this core group, surprisingly, also includes the CLR gene *CLEC12A*, whose role in interactions with microbes is still emerging, pointing to the possibility of as yet undefined, but important, roles for this CLR in microbial recognition. Beyond the shared signatures of positive selection across lineages, these core rapidly evolving PRRs also tended to have a higher number of sites predicted to be under positive selection, with many of the rapidly evolving amino acid residues falling into functionally relevant regions of these receptors, namely the extracellular carbohydrate-binding domains.

Outside of this core set of positively selected genes, we observed lineage-specific patterns of positive selection among the remaining PRRs. These different patterns of selection across the three mammalian lineages suggest the possibility that distinct populations of microbial species may have played a role in shaping the evolution of these mammalian receptors. Importantly, our analyses of the CTLDs of mammalian selectins revealed little evidence for positive selection in these genes with high levels of conservation across lineages. This further underscores the role of microbial pathogen interactions in driving the evolutionary signatures we observe across this gene set of PRRs.

### Rapidly Evolving Codons in Mammalian Langerin (CD207) Correspond with Amino Acid Positions at Key Ligand Recognition Interfaces

The set of PRRs under positive selection in all three of the tested mammalian lineages includes *Langerin (CD207)*, a CLR expressed primarily by the Langerhans cells of the skin as well as other professional antigen presenting cells. Langerin has an established role in the activation of critical inflammatory responses following direct detection of diverse microbial pathogens, including fungi, viruses, and bacteria (de Witte et al. 2007; de Jong et al. 2010; van der Vlist et al. 2011; van Dalen et al. 2019). In particular, Langerin has been shown to be able to recognize and bind to ß-glucans in *Candida* species as well as the skin-associated fungal species *Malassezia furfur* (de Jong et al. 2010). Bacterial recognition by Langerin has been observed for multiple species, including *Staphylococcus aureus*, a major cause of skin infections (Yang et al. 2015; van Dalen et al. 2019). In the context of both fungal and *S. aureus* infection, Langerin has been shown to play a role in regulating inflammatory Th17 responses (Sparber et al. 2018; van Dalen et al. 2019). Structural studies of human Langerin have revealed it to have a canonical CLR fold, with a Glu-Pro-Asn (EPN) motif in the primary ligand binding site, suggestive of a ligand preference for mannose and mannose-type carbohydrates (Tateno et al. 2010; Feinberg et al. 2011; Hanske et al. 2017). Interestingly, recent work examining the ligand-binding profiles of Langerin homologs from humans and mice identified distinct differences in the binding specificities for more complex bacterial-derived glycans among these homologs, despite conservation of the EPN motif in the binding site (Hanske et al. 2017). This suggests that sequence variation in the Langerin CTLD may play an important role in modulating microbial recognition.

**Fig. 2.**
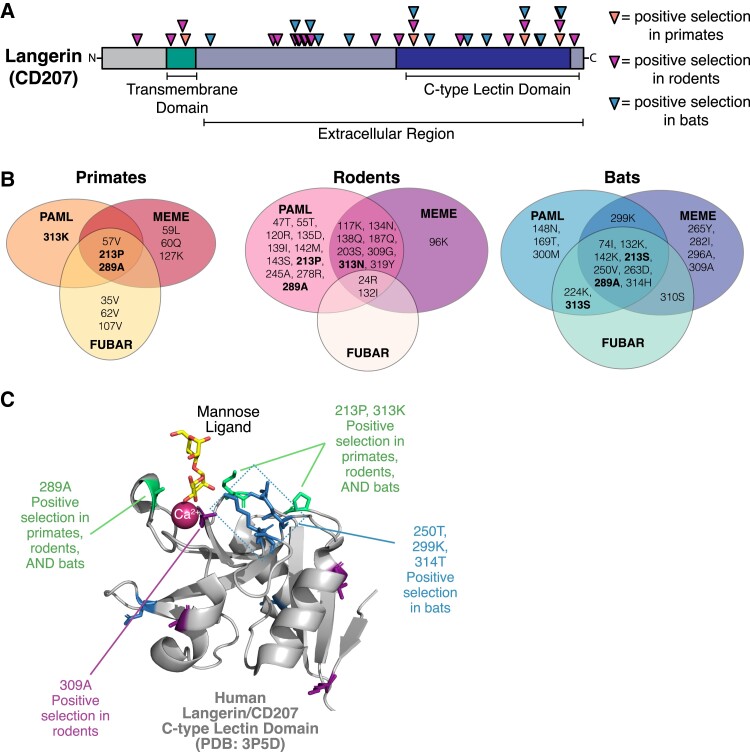
Diversification of Langerin (CD207) ligand-binding interfaces in all mammalian lineages. (*A*) Positively selected residues (triangles) predicted by PAML (Model 8, BEB > 0.9) cluster primarily in the extracellular portion of Langerin (CD207), with many in the CTLD. A number of positively selected sites in the CTLD are common across primates (orange triangles), rodents (purple triangles), and bats (blue triangles). (*B*) Agreement between different algorithms for identifying site-specific positive selection in Langerin of different mammalian groups. Listed residue numbers correspond to the position in the human Langerin sequence. Single letter residues correspond to the amino acid identity in human (primates, left), house mouse (rodents, middle), or black flying fox (bats, right) sequences. Bolded residues are those predicted to be under positive selection across all mammals by one or more tests. (*C*) Positively selected sites mapped onto a crystal structure of the human Langerin CTLD (gray, PDB:3p5*d*) in complex with a mannose ligand (yellow) and Ca^2+^ ion (magenta) (Feinberg et al. 2011). Positively selected sites in all three lineages (colored in green) along with several sites from rodent (blue) and bat (purple) analyses are shown with sidechains and surround the ligand binding site.

To determine whether the signals of rapid evolution that we observe in *Langerin* across mammalian lineages might functionally correlate with differences in ligand preference, we first mapped the sites under positive selection in each lineage to the annotated protein domains (fig. 2*A*). A large proportion of positively selected sites in all three lineages mapped to the extracellular region of the protein, with many falling into the CTLD itself, including several overlapping amino acid positions which were predicted to be under positive selection in all three mammalian lineages.

In addition to the PAML algorithm, we also used the HyPhy suite programs mixed effects model of evolution (MEME) and fast unbiased Bayesian approximation (FUBAR) to independently assess individual amino acid sites for elevated dN/dS values across the *Langerin* coding sequence (Murrell et al. 2012, 2013). Although MEME, like PAML, assesses patterns of episodic selection occurring on at least one branch of the phylogeny, the FUBAR algorithm can be used to identify sites under pervasive positive selection across an entire phylogeny. These additional analyses, thus, provide both confirmatory and complementary methods to PAML for assessing site-specific rapid evolution. Agreement between the three algorithms was high across all positively selected sites in Langerin (fig. 2*B*). In particular, amino acid positions 213 and 289, which were identified by PAML analyses in all three lineages, showed signatures of positive selection in the MEME and FUBAR analyses in both primates and bats. Similarly, multiple methods independently highlighted position 313 as rapidly evolving in bats and rodents, in agreement with the PAML analyses of primate sequences. Rapid evolution of other lineage-specific sites was also supported by all three analyses (fig. 2*B*).

The convergence of these signatures of rapid evolution on the Langerin CTLD and these three residues (213, 289, and 313) across multiple mammalian lineages hints at possible functional significance to amino acid changes at these positions. When mapped onto a crystal structure of the Langerin CTLD in complex with a mannose ligand and a coordinating calcium ion, we observed that many of the residues under positive selection clustered around the ligand binding site (fig. 2*C*). This supports the hypothesis that variation at these positions across mammalian Langerin homologs might result in differences in microbial glycan binding specificities. Furthermore, this suggests the possibility that the signals of rapid evolution we observe in mammalian *Langerin* homologs was driven by the selective pressure to maintain the ability to recognize specific microbial species through distinct microbial glycans on their surfaces and in their cell walls.

### Mapping Patterns of Substitution in an Invasive Aspergillosis Susceptibility Allele of *MelLec* (*Melanin Lectin/CLEC1A*) across Primates

Unlike many CLRs, which can recognize similar ligands present on many different species of microbes, MelLec (also known as CLEC1A), was recently identified as being a highly specific receptor for 1,8-dihydroxynaphthalene (DHN)-melanin, a critical component of the cell walls of a relatively limited group of fungal species (Stappers et al. 2018). Included in these DHN-melanin-producing fungi are the human fungal pathogens *Aspergillus fumigatus* and the black yeasts, which account for significant morbidity and mortality in both immune-suppressed and immunocompetent patients worldwide (Brown et al. 2012; Seyedmousavi et al. 2014). Recognition of DHN-melanin in fungal cells via MelLec has been demonstrated to be critical for the activation of an antifungal immune response and survival of systemic *A. fumigatus* infection in *in vivo* models. Notably, a common human polymorphism causing a single amino acid change (Gly26Ala, rs2306894) has been identified in the cytoplasmic region of the MelLec protein. This Ala26 allele has been associated with higher probability of invasive Aspergillosis in transplant patients and has also been shown to result in decreased production of critical cytokines in response to fungal stimulation in *in vitro* experiments (Stappers et al. 2018). Combined, these data support a role for *MelLec* in the immune responses to fungal infection in both mice and humans.

Our PAML analyses revealed signatures of recurrent positive selection in *MelLec* in both the primate and rodent lineages (fig. 1). Although significance by LRT varied for primate analyses of *MelLec* depending on whether a species or gene tree was used in the analysis, manual inspection of the alignments revealed extensive sequence variation at PAML-identified sites across the primate *MelLec* orthologs (see Methods and supplementary data file 1, Supplementary Material online). This suggests that interactions between these mammalian groups and pathogenic fungi may have played a role in shaping amino acid diversification in this PRR. Furthermore, the rapidly evolving amino acids within MelLec include several in the CTLD, consistent with the potential for sequence variation to confer changes in ligand-binding affinity or specificity among different MelLec homologs (supplementary data file 1, Supplementary Material online).

**Fig. 3.**
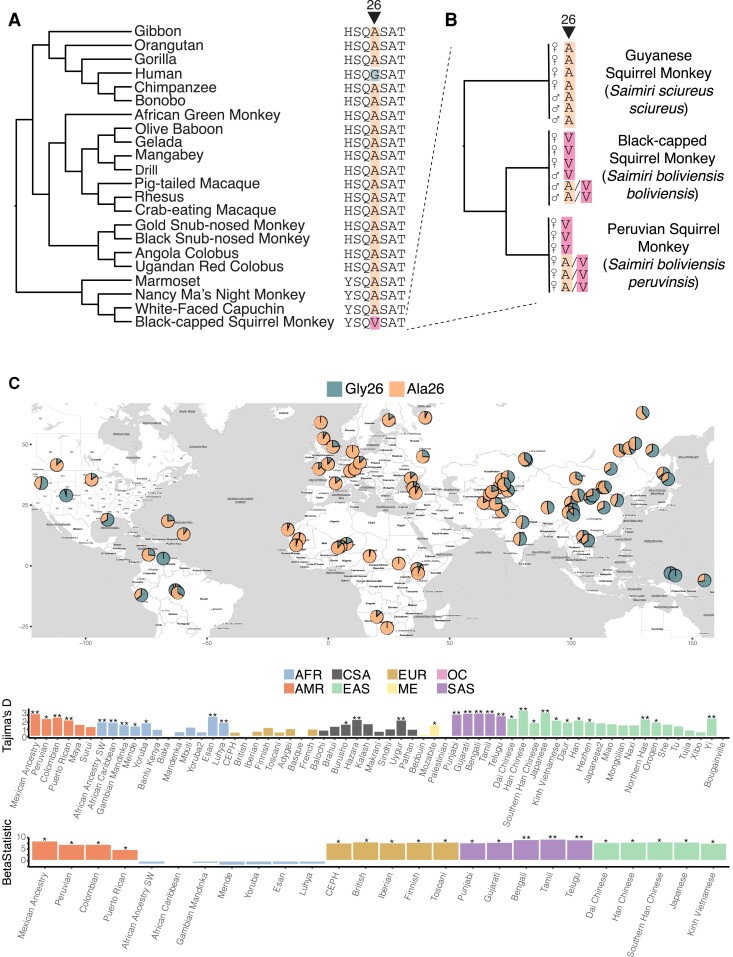
Single nucleotide polymorphisms in primate populations converge on a single site in *Melanin Lectin (CLEC1A)*. (*A*) Patterns of conservation and variation at amino acid position 26 of *MelLec* across primates. Most primate species carry the ancestral alanine allele (orange highlighting), whereas single nucleotide polymorphisms in both humans (glycine, green highlighting) and squirrel monkeys (valine, pink highlighting) confer missense mutations. (*B*) Genotypes of 19 unrelated squirrel monkey gDNA samples from three *S. boliviensis* subspecies. The sex and the amino acid identity at position 26 for each individual are indicated, with heterozygous individuals indicated as carrying both Ala and Val amino acids (A/V in Black-capped and Peruvian squirrel monkeys). (*C*) (top) Geographic distribution of the glycine 26 allele (green) at SNP rs2306894 in human populations. Allele frequencies are shown for populations from the 1KG Project and the HGDP. Individuals carrying the Ala26 allele (orange) have been previously shown to have higher risk of invasive fungal infections in stem-cell transplant patients (Stappers et al. 2018). (middle) Tajima's D values for populations from the HGDP and 1KG and (bottom) *β*^(1)^ for populations from the 1KG project showing evidence of balancing selection at the *MelLec* locus. For both plots, * empirical *P*-value < 0.05, ** empirical *P*-value < 0.01. Population abbreviations are as follows: AMR, America; AFR, Africa; EUR, Europe; CSA, Central-South Asia; ME, Middle East; SAS, South Asia; EAS, East Asia; OC, Oceania.

While mapping the positively selected sites in primate *MelLec* orthologs, we were surprised to find that at the site of the human polymorphism, Gly26, we observed a conserved alanine residue in all primates except humans and black-capped squirrel monkeys (*Saimiri boliviensis boliviensis*, fig. 3*A*). This suggests that Gly26 likely represents the derived human allele, while alanine is the ancestral allele among primates. Whether the alanine at position 26 in other primate homologs confers the same defects in cytokine production observed for the human allele is presently unknown. Although it is possible that sequence variation elsewhere in the primate MelLec homologs might compensate for the alanine at position 26, future experimental studies will be needed to assess how sequence variation at this and other sites contribute to function of the MelLec receptor.

We next explored the distribution of these two *MelLec* alleles in human populations. Across human populations in the 1000 Genomes Project (1KG) dataset, the frequency of the derived Gly26 allele varies widely, from only 0.11 in African (AFR) and 0.13 in European (EUR) populations to 0.65 in East Asian (EAS) populations (fig. 3*C*) (The 1000 Genomes Project Consortium 2015). Given the high frequency of the Gly26 allele in EAS populations, we turned to two additional resources to more comprehensively assess the distribution of this allele across Asia (GenomeAsia100K Consortium et al. 2019; Bergström et al. 2020). Using the Genome Asia 100K Browser and the Human Genome Diversity Project (HGDP), we observed that the Gly26 allele reached even higher frequencies in Oceanic (OC) and Southeast Asian (SAS) populations that were not represented in the 1000 Genomes dataset. The Gly26 allele was fixed in the populations from Papua New Guinea in the HGDP, though the sample size was small (*n* = 17) and at an allele frequency (AF) of 0.77 in PNG in the Genome Asia 100K dataset (*n* = 70) (fig. 3*C*). The HGDP also revealed a high frequency of the Gly26 allele in multiple American (AMR) populations (e.g., AF = 1 in Colombian, AF = 0.94 in Karitiana and AF = 0.95 in Pima), which may reflect the shared ancestry between native American and Asian populations. To quantify the allele frequency differences observed across these populations, we calculated pairwise F_ST_ between EUR populations (with low Gly26 frequencies) and the OC, SAS, and AMR populations and tested for significance relative to other single nucleotide polymorphisms (SNPs) on chromosome 12 (supplementary data file 1, Supplementary Material online). F_ST_ was high between all tested populations, falling in the tail of the empirical distributions, indicating an elevated signal of differentiation consistent with the allele frequency differences observed between these groups.

The extreme population differentiation of the rs2306894 Gly26Ala SNP could reflect that this locus has been a target of selection in human populations. Both positive and balancing selection can affect population differentiation and F_ST_ values. We first assessed whether rs2306894 or any other SNPs in *MelLec* showed signatures of local positive selection. Both searches of published scans for recent positive selection focusing on Asian populations as well as our own analysis of the Colombian population from the 1KG database using Relate showed no evidence for positive selection in *MelLec* in human populations (supplementary fig. S2, Supplementary Material online) (Voight et al. 2006; Liu et al. 2017; Speidel et al. 2019, 2021). Next, we calculated Tajima's D in 1 kb windows across all of Chromosome 12 in each population from the HGDP and 1KG datasets. Notably, we observed elevated Tajima's D values for the window containing *MelLec* and rs2306894 in the majority of the tested populations, with a significantly positive value in 31 of 62 populations assessed (empirical *P* < 0.05), suggestive of balancing selection acting at this locus (fig. 3*C*, middle). To further confirm this, we ran BetaScan, a more sensitive method for detecting balancing selection, where high *β*^(1)^ statistics are indicative of an excess of SNPs at similar frequencies, a key feature of genomic regions under balancing selection (Siewert and Voight 2017, 2020). The *β*^(1)^ statistic was significantly elevated (empirical *P* < 0.05) for *MelLec* in all of the 1KG populations except for the AFR populations, further suggesting that this gene has been subject to balancing selection in many human populations (fig. 3*C*, bottom).

It is important to note that while previous functional studies have focused solely on the Gly26Ala SNP, our analyses revealed that this SNP is in perfect linkage disequilibrium (LD) with a large number of other SNPs within *MelLec* (e.g., 42 SNPs in *r*^2^ = 1 with rs2306894 in EAS, spanning 8 kb) making it challenging to distinguish the target of the selective signatures we identify here (supplementary data file 1, Supplementary Material online). The vast majority of these SNPs fall into intronic regions and are documented eQTLs for *MelLec* in multiple tissues in the Genotype-Tissue Expression (GTEx) project (Lonsdale et al. 2013). Two of these SNPS in LD with rs2306894 fall within regulatory regions which could have direct regulatory effects on expression of *MelLec*: rs2306893 in the 5′UTR and rs2277416 in a splice region. Future studies probing the effects of these SNPs on MelLec function may further our understanding of how they individually or collectively contribute to fungal disease and reveal a more nuanced understanding of the target of the balancing selection signatures we observe.

Beyond humans, we also noted that the black-capped squirrel monkey sequence from the NCBI GenBank database carried a valine at position 26, in contrast to the alanine of all other primates (fig. 3*A*). To confirm this observation and investigate the patterns of substitution at this position among squirrel monkey populations, we amplified the region surrounding this SNP from multiple genomic DNA (gDNA) samples from black-capped squirrel monkeys (*S. boliviensis boliviensis*) as well as two other closely related squirrel monkey subspecies: Peruvian squirrel monkeys (*S. boliviensis peruvinsis*) and Guianan squirrel monkeys (*S. sciureus sciureus*). In total, we genotyped 19 unrelated individuals from these three subspecies. Interestingly, the Guianan squirrel monkeys were universally homozygous for the ancestral Ala26 allele, whereas no individuals homozygous for this allele could be found in the other two subspecies (fig. 3*B* and supplementary fig. S3, Supplementary Material online). Among black-capped and Peruvian squirrel monkeys, there was a mix of individuals homozygous for the derived Val26, as well as heterozygous individuals, again raising intriguing questions about the potential selective pressures that have shaped allele frequency distributions in squirrel monkeys as in humans.

To rule out the possibility that the lack of observed sequence variation in other primates might be due to sampling bias of the publicly available sequences in GenBank, we also looked for variation at this locus among hominoid primates using data from the Great Ape Genome Project (Prado-Martinez et al. 2013). There was no evidence in these data for any sequence variation at amino acid position 26 in gorillas, bonobos, chimpanzees, or orangutans (supplementary data file 1, Supplementary Material online). Combined, these data strongly suggest that mutation of this locus has occurred independently in humans and squirrel monkeys, perhaps due to similar evolutionary pressures in these species from fungi or other microbial species.

### Extensive Positive Selection across *CLEC12A* in Primates, Bats, and Rodents Portends an Unidentified Role in Microbial Recognition and Binding

**Fig. 4.**
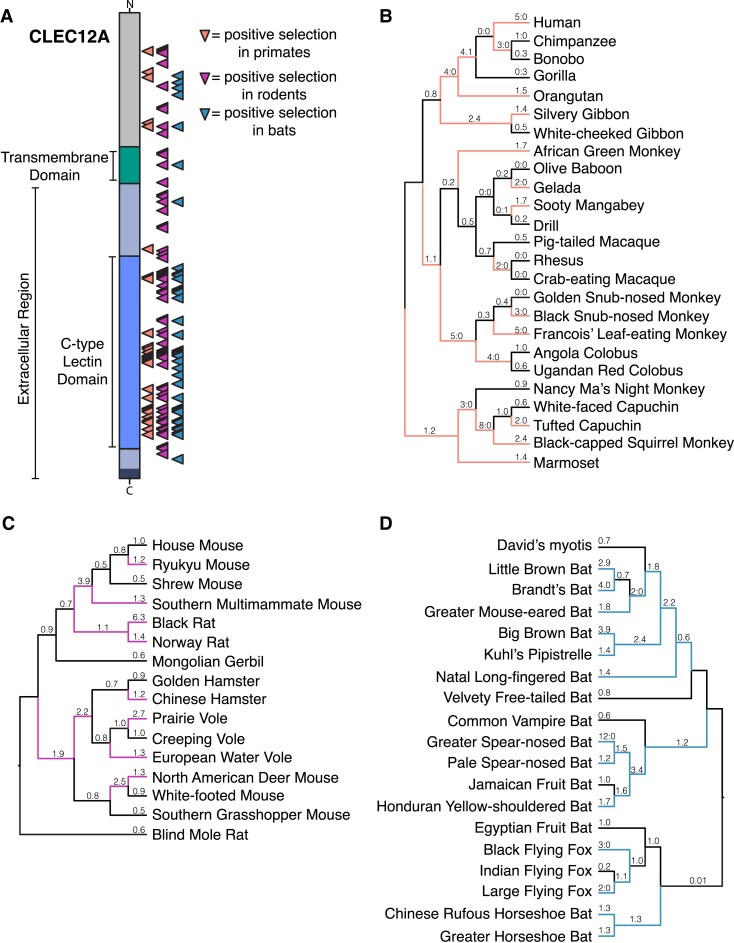
Extensive positive selection in *CLEC12A* across mammals reveals a new host–pathogen battleground. (*A*) Diagram showing sites under positive selection in CLEC12*A* in primates (orange triangles), rodents (purple triangles) and bats (blue triangles). Indicated sites were predicted by PAML (Model 8, BEB > 0.9). Locations of the CTLD and transmembrane domain are indicated on the left. (*B*)–(*D*) dN/dS values for *CLEC12A* were calculated across the species phylogenies of primates (*B*), rodents (*C*), and bats (*D*) using PAML (free ratios, Model = 1 setting). Lineages with elevated dN/dS values (>1), suggestive of positive selection along that branch, are indicated with colored lines. Calculated dN/dS values are listed above each branch and for branches lacking either nonsynonymous or synonymous sites; ratios of the respective substitution numbers (N:S) are indicated.

In addition to genes with well-established roles in immune responses to microbial pathogens, our analyses also revealed extensive positive selection occurring at sites within the *CLEC12A* gene, a more mysterious member of the CLR family of receptors. Originally identified as a receptor for uric acid, a marker of cell death, other reports have identified roles for this receptor in the recognition of hemozoin produced by *Plasmodium* spp. during infection as well as in the regulation of antibacterial autophagy responses (Neumann et al. 2014; Begun et al. 2015; Raulf et al. 2019). Most recently, CLEC12A, has been shown to directly bind to a number of gut-resident bacteria and is required for the phagocytosis of these bacteria and subsequent modulation of microbiome community composition (Chiaro et al. 2023). Although the exact moiety that CLEC12A engages remains undefined, these data strongly suggest the possibility that CLEC12A is also capable of recognizing molecular patterns found in the bacterial cell wall, including bacterial glycans. Given the breadth of the currently known ligands and roles of CLEC12A and its expression predominantly in myeloid cells, it is likely that the full scope and nature of the interfaces between CLEC12A and pathogenic microbes has not yet been revealed. Further supporting this idea, our phylogenetic analyses of *CLEC12A* revealed strong signals of positive selection on this gene across all mammalian lineages, suggestive of strong selection imposed on this gene by interactions with, perhaps, diverse pathogens (fig. 4). In fact, in both bats and primates, the gene-wide dN/dS calculated by PAML was >1 (supplementary data file 1, Supplementary Material online). *CLEC12A* was the only gene analyzed in this study for which this was true and supports the model that *CLEC12A* is evolving under remarkably strong positive selection in mammals.

Although positively selected sites were distributed across the entire coding sequence of *CLEC12A*, a large number fall directly in the CTLD, a pattern which is most pronounced in primates (orange triangles, fig. 4*A*). Many of these sites were independently predicted to be rapidly evolving by PAML, MEME and FUBAR and tend to cluster in the same regions in all three mammalian groups, suggesting these may be regions important for the immune or ligand binding functions of the protein (supplementary data file 1, Supplementary Material online). Given the large number of sites under positive selection in the CTLD, no discernable patterns emerged from mapping these sites onto AlphaFold-predicted structures of CLEC12A CTLD homologs from different species that might hint at effects of sequence diversification on ligand binding. Of note, however, was the fact that despite the primary sequence divergence across mammals, there were no significant differences in the AlphaFold-predicted structures of primate, rodent and bat homologs suggesting that more subtle modifications in structure may underlie any functional differences between homologs (supplementary fig. S4, Supplementary Material online).

To identify specific rapidly evolving branches in each mammalian lineage, we applied models implemented in PAML that allow calculation of dN/dS for each branch of a given phylogenetic tree (fig. 4*B*–*D*). This temporal view of the evolution of *CLEC12A* revealed extensive episodic positive selection across each of the mammalian phylogenies. Among the simian primates, all three major groups (Hominids, Old World, and New World Monkeys) contained branches with elevated dN/dS values, though these values were slightly higher among both the ancient and recent branches in the hominid and New World Monkey lineages (fig. 4*B*). Similar patterns can be seen in the rodent and bat phylogenies, where positive selection was also rampant (fig. 4*C* and *D*). Consistent with the elevated gene-wide dN/dS value observed for bat *CLEC12A* (dN/dS = 1.2, supplementary data file 1, Supplementary Material online), especially high substitution rates were abundant across the bat phylogeny, and in particular among the new world leaf-nosed bats (*Phyllostimidae*), a group which includes the spear-nosed bats, Jamaican fruit bat and the Honduran yellow-shouldered bat (fig. 4*D*). Combined, the strength of the signals of rapid evolution that our analyses revealed in *CLEC12A* across multiple mammalian lineages, suggest it functions as an underappreciated but critical component in the arsenal of immune receptors that engage with microbial pathogens and play a role in immune defenses against infection. Although it is theoretically possible that the signals we observe in *CLEC12A* have been driven by already identified ligands and interactions, we hypothesize that there are likely undiscovered interactions between CLEC12A and other microbial species for which this sequence variation will have functional implications.

## Discussion

Our study revealed widespread signatures of rapid evolution across glycan-recognition PRRs in three major mammalian lineages: primates, rodents, and bats. Such strong signatures of positive selection are frequently associated with host–pathogen arms races, signifying the consequential impacts on fitness associated with these interactions. We hypothesize that the evolutionary signatures we observe among CLRs and related factors represent a new axis in these arms races where hosts keep pace with the numerous and well-studied evasive strategies microbes use to prevent detection of their immunogenic glycan-rich surfaces. Consistent with this hypothesis, we found that positive selection among these genes is often enriched in functionally significant portions of the protein, namely in the CTLDs which directly interact with glycans. In Langerin, this pattern was particularly clear, with a cluster of rapidly evolving residues falling directly surrounding the ligand binding pocket of the CTLD (fig. 2*C*). Positively selected sites in Langerin include amino acid position 313, which has previously been determined to contribute significantly to ligand binding, with mutations at this position resulting in a complete lack of recognition of certain simple carbohydrate ligands (Tateno et al. 2010). Across all the mammalian species we analyzed in this study, we observed eight different amino acids sampled at this position, a finding that strongly points to functional differences in ligand binding and specificity.

The finding that the highly specific DHN-melanin binding MelLec receptor is rapidly evolving in both primates and rodents is particularly exciting. To date, studies of host–microbe evolutionary arms races have largely involved only interactions with viruses or bacteria; the role of eukaryotic pathogens, such as fungi, in shaping the evolution of mammalian host species has remained unexplored. Rapid evolution in *MelLec* across species when paired with the emerging patterns of substitution at a functionally important site in both humans and squirrel monkeys strongly suggests that fungi can, in fact, play an important role in shaping the evolution of mammalian immune systems. Additionally, many of the other PRRs identified as rapidly evolving in this study also engage with fungal pathogens, suggesting that the breadth of host proteins shaped by interactions with pathogenic fungi may be extensive.

Our population genetics analyses of the human *MelLec* Gly26Ala SNP further revealed strong population differentiation in the allele frequencies of this SNP along with signals of balancing selection within this locus in many human populations. This raises several intriguing hypotheses: first, that different association with fungal species across geographic regions might partially account for the allele frequency differences observed across human populations. Other factors, such as lifestyle and/or dietary differences across human populations could also play a role in driving the population differentiation we observe. Whether and how these different pressures shaped the distribution of these *MelLec* alleles in human populations remains a fascinating challenge to dissect. A second hypothesis that arises from our population genetic analysis of *MelLec* suggests that although the Gly26 allele appears to be protective under some circumstances, there may be tradeoffs associated with changes at this position, reflected in the maintenance of the ancestral Ala26 allele in human populations and the signals of balancing selection we observe. Indeed, although MelLec is essential for protection against invasive disease caused by fungal species like *A. fumigatus*, its function was shown to be detrimental in *in vivo* models of asthma driven by the same fungal species suggesting that MelLec activity has a complex impact on establishing appropriate immune responses to fungi (Stappers et al. 2018; Tone et al. 2021). Whether and how mutation of position 26 (or other sites) within the *MelLec* locus might contribute to these differing outcomes remains to be seen but may provide some insight into the signals of balancing selection we observe in this gene.

Previous analysis of carbohydrate-ligand binding in different mammalian Langerin homologs led to the surprising finding that although specificity in ligand binding for simple carbohydrates was similar across different Langerin variants, dramatic differences were observed in the context of complex carbohydrates and intact bacterial cells (Hanske et al. 2017). These differences were identified despite high conservation in the solved crystal structures of the CTLDs from these homologs, suggesting that more subtle structural or sequence variation underlies variability in ligand binding. Our analyses of the *CLEC12A* gene suggest this may be a general feature among these rapidly evolving CLRs. In CLEC12A, we observed extensive diversification of the primary sequence in all mammalian lineages analyzed, but very little change in the predicted structures of diverse variants of this protein (supplementary fig. S4, Supplementary Material online). This suggests that the CLR fold is highly robust to sequence variation and underscores the need for future studies to parse the functional implications of the sequence variation we observe.

Our results raise intriguing questions about the interactions that drive rapid evolution in glycan-recognition receptors and what the tradeoffs may be for interactions with other microbes. Many of these PRRs are nonspecific, involved in the recognition of many diverse glycan structures found in multiple microbial species. This suggests that diversification of the carbohydrate recognition domains of these PRRs could have a profound impact on the recognition of numerous microbial species. Although this may make it challenging to identify the exact molecular changes or microbial species that have driven rapid evolution in these glycan PRRs, this system represents a unique opportunity to study the tradeoffs associated with rapid evolution, a topic that has been largely ignored in protein–protein arms races, where the focus has remained on 1:1 interactions between host proteins and highly specific pathogenic substrates. Recent advances in high-throughput profiling of host lectin interactions with complex microbial glycans when applied to these rapidly evolving PRRs will likely help to shed light on these questions of what drove these signals of evolution and what the consequences might be for specific microbial recognition (Stowell et al. 2014; Jégouzo et al. 2020).

Finally, our phylogenetic screen identified extensive positive selection among rodent and, in particular, bat glycan PRRs, where a striking 81% of the genes we analyzed were found to be rapidly evolving. This suggests that for these carbohydrate-recognition receptors, evolution has been driven by lineage-specific microbial communities, perhaps including both pathogenic and commensal species. Combined, our data reveal a new axis of evolutionary arms races—involving microbial glycan detection—and dramatically expand the realm of host–microbe interactions to include fungal pathogens with consequential influence on the evolution of eukaryotic biology.

## Materials and Methods

### Phylogenetic Analyses

Candidate gene ortholog sequences were obtained from NCBI GenBank either through gene name searches or by BLAST searches using the Human ortholog sequence as query (see supplementary data file 1, Supplementary Material online for full list of accession numbers). Additional BLAST searches were carried out using alternate species as query to confirm that the same subsets of genes were being identified through different searches. Orthologous relationships between genes were further confirmed by phylogenetic and synteny analysis and species were excluded from evolutionary analysis if clear orthology could not be established. Phylogenetic tree analysis of some of the more divergent genes, like *CLEC12A*, confirmed that orthologs of *CLEC12A* from all three mammalian groups cluster together on a single branch, removed from the other CLR genes (supplementary fig. S1*A*, Supplementary Material online).

Sequences were obtained for all available simian primate species, *Myomorpha* species (minus *Jaculus jaculus*, for which we could not consistently find well-annotated orthologs), and the *Chiroptera*. Coding sequences were downloaded and aligned using the Geneious Translation Align function with the MUSCLE algorithm option. Alignments were manually inspected and trimmed to remove gaps, ambiguous regions of the alignment and stop codons. Alignments were used to construct gene trees using IQ-TREE and the GTR + G + I model with 100 nonparametric bootstraps (Nguyen et al. 2015). Both gene trees and generally accepted species phylogenies for each of the mammalian groups were used for downstream evolutionary analyses. Alignments and trees used in analysis can be found in supplementary data file 2, Supplementary Material online. Data shown in figure 1 are based on analyses done with species trees, but all of the results of the analyses can be found in supplementary data file 1, Supplementary Material online. Unless otherwise noted, all computational analysis was performed using the University of Utah Center for High Performance Computing.

Positive selection was assessed using the codeml function of the PAML software package (v4.9) with the F3 × 4 codon frequency model (Yang 2007). Gene-wide dN/dS values were calculated using model 0. To test whether a subset of amino acid sites were evolving under positive selection, we performed LRTs, comparing pairs of NSsites models including: M1 (neutral evolution) versus M2 (positive selection) and M7 (neutral, beta distribution dN/dS ≤ 1) versus M8 (positive selection, beta distribution allowing for dN/dS > 1). For genes with statistical support for positive selection, specific amino acid positions were identified as being under positive selection based on having a Bayes Empirical Bayes (BEB) posterior probability of greater than 90% in the M8 model. For the free ratios analysis of *CLEC12A*, codeml Model 1, allowing variation of dN/dS across branches of the phylogeny, was run on the *CLEC12A* alignments with an unrooted species tree for each lineage.

The BUSTED, MEME, and FUBAR programs from the HyPhy suite (version 2.5.41) were run through the command line with the same input alignments and trees used for PAML analyses and default options (Pond et al. 2005; Murrell et al. 2012, 2013, 2015). Results were visualized using the HyPhy Vision web server. For several of the BUSTED analyses, we noticed that the algorithm found statistically significant support for positive selection in alignments that had very high levels of conservation determined by other methods (e.g., Primate *FIBCD1* and *Dectin1*). When we examined these results, we found that the signal was being driven entirely by codons containing multiple nucleotide substitutions, which has been a documented confounding variable in branch-site models of rapid evolution (Venkat et al. 2018). For these anomalous results, we re-ran the analyses without these multiply substituted sites and found that these genes were no longer predicted to be under positive selection by BUSTED (see “BUSTED *P*-value with MNMs removed” column in supplementary data file 1, Supplementary Material online). These re-runs are reflected in the results displayed in figure 1.

Codon alignments of the Human CTLDs from each of the CLRs in the gene set were used as input to IQ-TREE for phylogenetic tree construction (fig. 1*B*) (Nguyen et al. 2015). The VT + G4 substitution model was selected as the best fit model by the ModelFinder function, and 100 nonparametric bootstrap replicates were performed (Kalyaanamoorthy et al. 2017). Some IQ-TREE analyses were performed with the IQ-Tree webserver (Trifinopoulos et al. 2016). CTLDs were identified based on annotated domains from UniProt and genes with multiple CTLDs (e.g., *MRC1* and *MRC2*) were excluded. An alternate version of this tree built from an alignment of nine representative species spanning all three mammalian groups assessed is shown in supplementary figure S1*B*, Supplementary Material online. Species included were: *Homo sapiens, Mucaca mulatta, S. boliviensis, Mus musculus, Microtus ochrogaster, Nannospalax galili, Myotis myotis, Pteropus alecto,* and *Rhinolophus sinicus*. Tree topology varied only slightly across species and in this pan-species tree.

### *MelLec* Human Population Genetics Analyses

To map the geographic distribution of the G26A polymorphism (rs2306894) in Human *MelLec (CLEC1A)*, sampling locations of 1KG on GRCh38 and HGDP populations were downloaded from the International Genome Sample Resource (Zheng-Bradley et al. 2017; Lowy-Gallego et al. 2019; Bergström et al. 2020). Chromosome 12 VCF files for HGDP and 1KG datasets were downloaded from their respective FTP sites (see Data Availability statement below). VCFtools was used to obtain the allele frequency at G26A for all populations, and the map was created using the R library ggmap (Danecek et al. 2011).

Tajima's D was calculated using VCFtools and *β*^(1)^ statistics using BetaScan2 (Siewert and Voight 2020). The derived allele was obtained from ancestral FASTA files downloaded from Ensembl (see Data Availability statement below). Empirical *P*-values were calculated in R by comparison with all other test statistic values on chr12 and plots were generated with ggplot2 (R Core Team 2022). Cowplot was used to combine the map, Tajima's D, and *β*^(1)^ plots.

r^2^ was calculated between rs2306894 and SNPs within 100 kb in either direction to identify pairs in high linkage disequilibrium using VCFtools and plink2 (Chang et al. 2015). We also generated a population-specific chromosome 12 VCF, using VCFtools, from the 1KG Colombian population to test for positive selection using Relate v1.1.8 and the add-on module for selection, which infers how quickly a mutation spread through the population based on genome-wide genealogies (Speidel et al. 2019, 2021).

### Squirrel Monkey gDNA MelLec Genotyping

Squirrel monkey gDNA was originally isolated from blood samples kindly provided by the MD Anderson Squirrel Monkey Resource and Breeding Center in September 2015. The provided samples came from unrelated individuals and additional information including Sample IDs, sex and age of the animals can be found in supplementary figure S3, Supplementary Material online. One additional gDNA sample from *S. sciureus sciureus* was isolated from the AG05311 fibroblast cell line provided by the Coriell Institute. All gDNA samples have been stored at −20 °C.

Primers MS_B17 and MS_B20 were designed to amplify a ∼500 bp fragment including the entirety of Exon 1 of *MelLec (CLEC1A)* which contains the polymorphic site (amino acid 26), along with flanking sequence. The black-capped squirrel monkey genome saiBol1was used as a reference for primer design. Polymerase chain reactions were performed using Phusion Flash polymerase and 50 ng of each gDNA sample from the squirrel monkey individuals. PCR products were confirmed on a gel, purified with Exo-SAP and Sanger sequenced at the University of Utah Sequencing Core using primer MS_B19. Genotypes were called based on visualization of Sanger sequencing traces in Geneious. Primer sequences are as follows:

MS_B17 TCCATGAGAGGTGCAAACAG
MS_B20 AGTTGTGGAAAGCGCACAG
MS_B19 ACATGCTGTTTCCCTTCAGC

### Structural Modeling and Comparisons of CLEC12A CTLDs

The structures of the CTLDs of nine mammalian CLEC12A orthologs were modeled using AlphaFold (v 2.1.2) (Jumper et al. 2021). The predicted structure with the highest confidence (ranked_0.pdb) for each ortholog was compared with all other species using jFATCAT through the RCSB PDB Pairwise Structure Alignment tool (Prlić et al. 2010; Burley et al. 2018; Li et al. 2020). Alignments were performed using both the rigid and flexible alignment algorithms and results were identical between the two. RMSD values were plotted as a heatmap in R (supplementary fig. S4, Supplementary Material online). All ranked_0 predicted structures and CTLD sequences used for modeling can be found at: dx.doi.org/10.6084/m9.figshare.23535738.

## Supplementary Material

evad119_Supplementary_Data

## Data Availability

NCBI accession numbers for all genes analyzed are provided in supplementary data file 1, Supplementary Material online. Alignments and trees used in positive selection analyses are provided in supplementary data file 2, Supplementary Material online. Genotypes for great ape species at the position of the rs2306894 human polymorphism were obtained from: https://www.biologiaevolutiva.org/greatape/data.html. For analyses of *MelLec* in human populations, the following links were used to download or access the relevant datasets: Sampling locations: https://www.internationalgenome.org/data-portal/population. HGDP Chr12: https://ngs.sanger.ac.uk/production/hgdp/hgdp_wgs.20190516/. 1KG Chr12: http://ftp.1000genomes.ebi.ac.uk/vol1/ftp/data_collections/1000G_2504_high_coverage/working/20201028_3202_phased/. Ancestral FASTA files for GRCh38 (homo sapiens ancestor GRCh38.tar.gz downloaded March 2023): https://ftp.ensembl.org/pub/current_fasta/ancestral_alleles/. eQTL analysis available from GTEx: https://gtexportal.org/home/snp/rs2306894. F_ST_, Tajima's D, *β*^(1)^ statistics, and statistics from linkage disequilibrium analysis are provided in supplementary data file 1, Supplementary Material online. AlphaFold-modeled CLEC12A CTLD structures can be found on figshare at: dx.doi.org/10.6084/m9.figshare.23535738.
