# Supplementary material for: Rapid Evolution of Glycan Recognition Receptors Reveals an Axis of Host–Microbe Arms Races beyond Canonical Protein–Protein Interfaces
Source: Genome Biol Evol. 2023 Jun 30;15(7):evad119. doi: 10.1093/gbe/evad119 (PMC10329266; doi:10.1093/gbe/evad119)
Supplement: evad119_Supplementary_Data [file evad119_supplementary_data.zip › SupplementalFigures_Revised.pdf]

Supplemental Figures

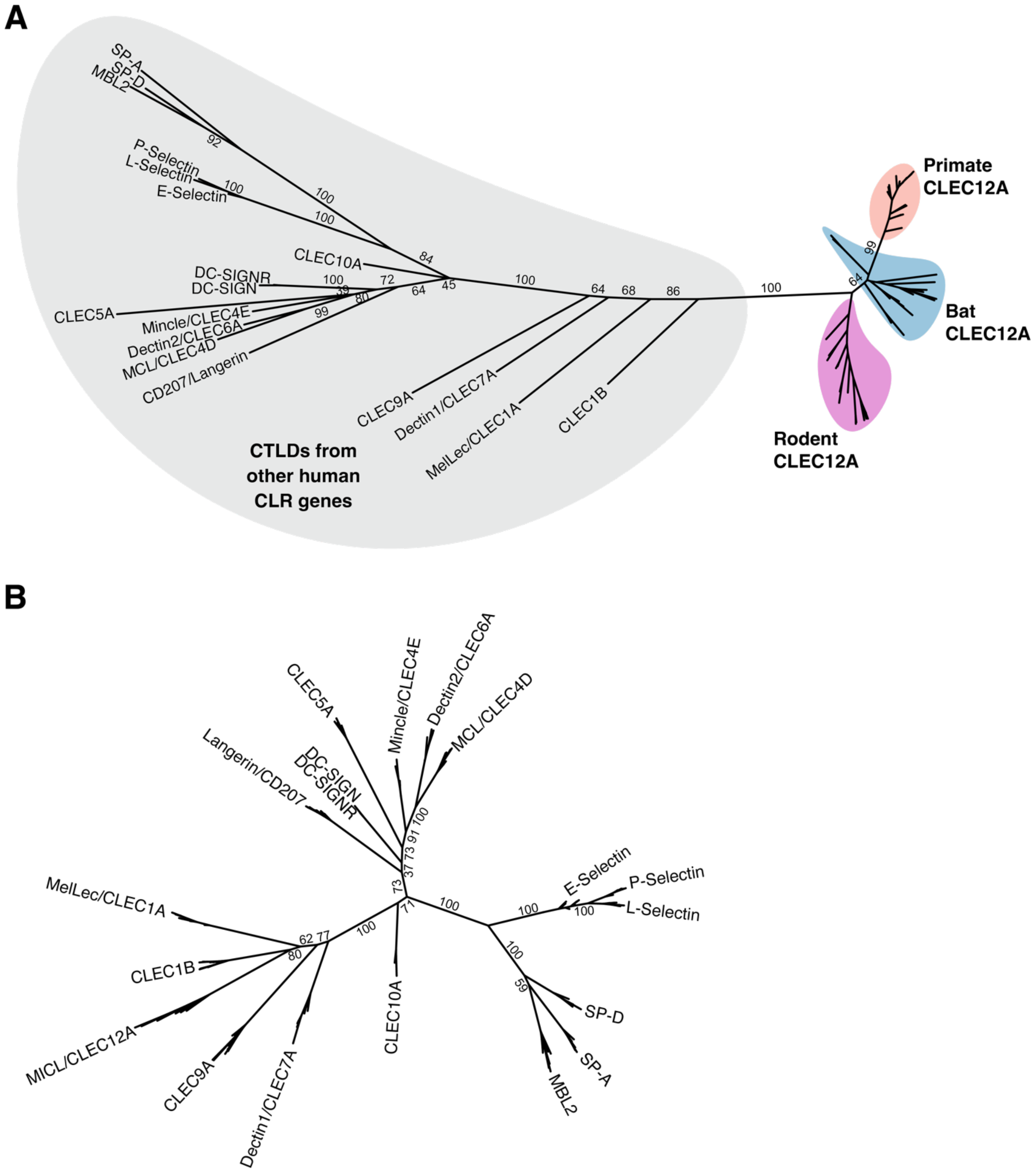

Supplemental Figure 1. **Phylogenetic analysis of mammalian C-type lectin receptor genes.** (A) Phylogenetic tree of the CTLDs of all analyzed genes from humans along with all of the analyzed CTLDs from the *CLEC12A* genes of primate, bat and rodent species. *CLEC12A* sequences cluster according to mammalian lineage (colored, highlighted branches) on a separate branch of the phylogeny from all other CTLDs. (B) Phylogenetic tree of the CTLDs of all analyzed genes made from an alignment of sequences from nine mammalian species from across all three lineages. Compare to Figure 1B. For both phylogenies, numbers indicate bootstrap values from tree construction using IQ-Tree.

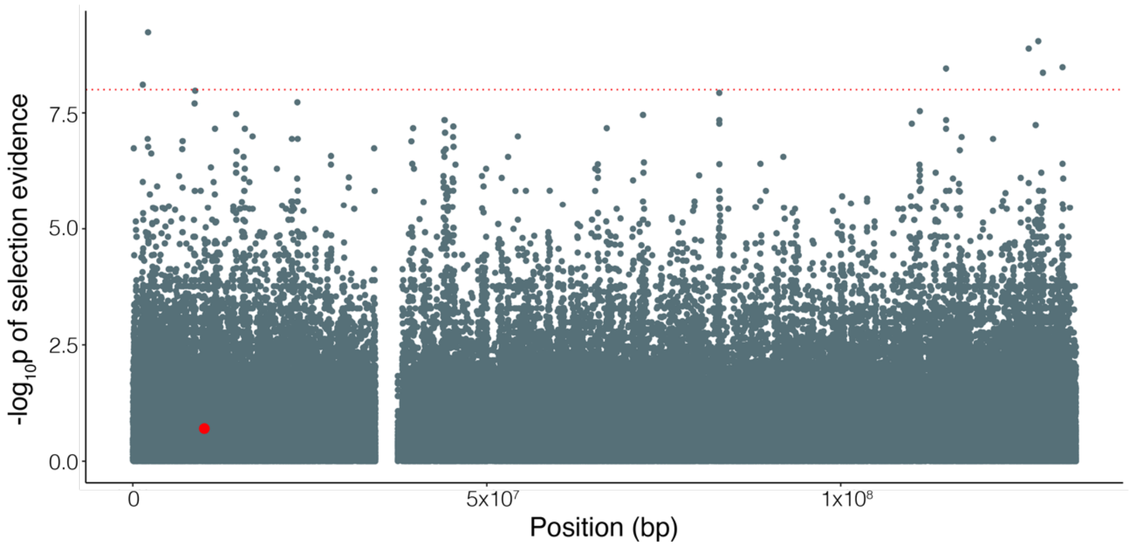

Supplemental Figure 2. **No evidence for positive selection in *MelLec* in the Colombian population.** Relate test for positive selection across chromosome 12 in the Colombian population from the 1KG database. The y-axis shows the -log10 p-value for selection evidence output by Relate, and the x-axis is the position in bp along chromosome 12. The dashed red line indicates the p-value for genome-wide significance. The red dot indicates the position of the *MelLec/CLEC1A* polymorphism of interest (rs2306894, Gly26Ala).

A

Species	Sample ID	Sex	Age	Genotype
Saimiri boliviensis boliviensis	4847	F	7	A/A
	4461	F	9	A/A
	92186	M	21	A/G
	5052	F	6	A/A
	3280	M	15	A/G
	3050	M	16	A/A
Saimiri boliviensis peruvinsis	2120	F	23	A/G
	3451	F	14	A/A
	4083	F	11	A/G
	4309	F	10	A/A
	2900	F	17	A/G
	3204	F	15	A/A
Saimiri sciureus sciureus	5779	F	2	G/G
	4738	F	8	G/G
	6503	M	9	G/G
	4113	F	11	G/G
	6894	M	9	G/G
	6505	M	9	G/G
	AG05311	F	Unknown	G/G

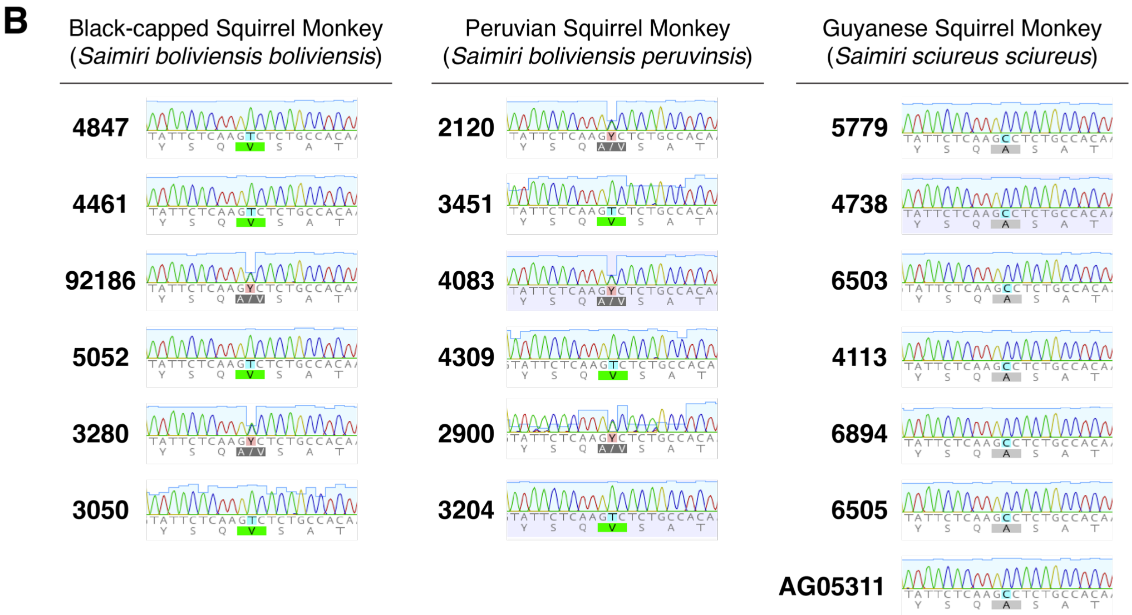

Supplemental Figure 3. Squirrel Monkey sample information and sequencing traces for *MelLec* SNP characterization. (A) Sample ID numbers, sex and age information for each of the 19 squirrel monkey samples that were genotyped for the *MelLec* SNP at amino acid position 26. Nucleotide genotype calls for each sample are indicated. (B) Sanger traces for sequencing of the *MelLec* Exon 1 from Squirrel Monkey samples. Sample IDs are given along with the region immediately surrounding the *MelLec* SNP (shaded in blue or red and indicated by the changing amino acid sequence below). Sequencing traces were aligned to the genomic sequence of *MelLec* obtained from the SaiBol1 genome on Ensembl. For *MelLec*, the coding strand is the chromosomal antisense strand so genotype calls in (A) are the complement of what is shown in the Sanger traces in (B) to match the nomenclature in human genotyping calls.

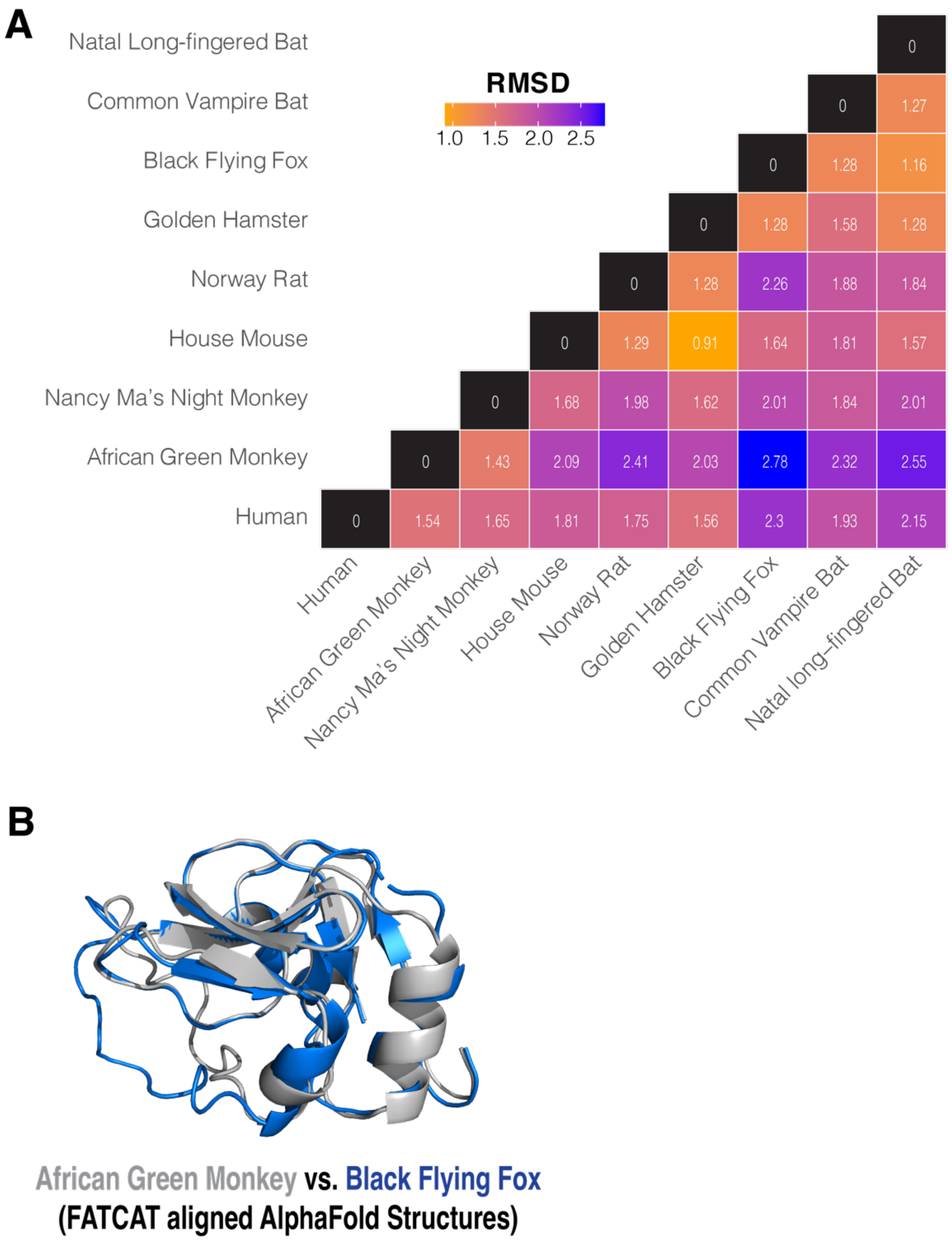

Supplemental Figure 4. Structural alignments of CLEC12A AlphaFold predicted CTLD structures reveal no significant differences in structures across species. (A) Heatmap indicating the pair-wise RMSD for CLEC12A CTLD structures predicted by AlphaFold. RMSD was calculated using the FATCAT structural alignment algorithm. RMSD values range from 0.91 Å(yellow, House Mouse vs. Golden Hamster) to 2.78 Å (blue, African Green Monkey vs. Black Flying Fox) but are all less than 3 Å, indicating high overall structural similarity. (B) FATCAT structural alignment of African Green Monkey (gray) and Black Flying Fox (blue) predicted structures. The overlay indicates high structural similarity with a few regions of disagreement in the foreground alpha helices.
